# Successful treatment of noninfectious hemorrhagic cystitis with intravesical hyaluronic acid in a 5-year-old boy: a case report

**DOI:** 10.1093/omcr/omag133

**Published:** 2026-07-27

**Authors:** Zlatan Zvizdic, Asmir Jonuzi, Mubina Hakulija, Semir Vranic

**Affiliations:** Department of Pediatric Surgery, Clinical Center University Sarajevo, Bolnička 25, 71000 Sarajevo, Bosnia and Herzegovina; Medical Faculty, University of Sarajevo, Čekaluša 90, 71000 Sarajevo, Bosnia and Herzegovina; Department of Pediatric Surgery, Clinical Center University Sarajevo, Bolnička 25, 71000 Sarajevo, Bosnia and Herzegovina; Medical Faculty, University of Sarajevo, Čekaluša 90, 71000 Sarajevo, Bosnia and Herzegovina; Medical Faculty, University of Sarajevo, Čekaluša 90, 71000 Sarajevo, Bosnia and Herzegovina; College of Medicine, QU Health, Qatar University, PO Box 2713, Doha, Qatar

**Keywords:** Hemorrhagic cystitis, pediatric hematuria, hyaluronic acid, intravesical therapy, glycosaminoglycan layer

## Abstract

Hemorrhagic cystitis (HC) in children is most associated with infection or treatment-related toxicity, whereas other cases remain uncommon. We report a 5-year-old boy presenting with recurrent painless gross hematuria in whom a structured, stepwise evaluation excluded glomerular, infectious, structural, and systemic causes. Cystoscopy revealed diffuse mucosal inflammation with focal hemorrhagic changes involving the bladder neck and posterior wall, consistent with noninfectious HC. In the absence of an identifiable etiology and with persistent symptoms, management was directed toward restoring urothelial barrier integrity using intravesical hyaluronic acid (HA). The clinical response was rapid, with resolution of gross hematuria after the first instillation, followed by sustained remission and progressive endoscopic improvement. The treatment was well tolerated, and the patient remained disease-free at 12 months of follow-up. This case highlights the diagnostic complexity of unexplained hematuria in children and supports a potential role of urothelial barrier dysfunction in the pathogenesis of noninfectious HC.

## Introduction

Hemorrhagic cystitis (HC) is characterized by bleeding originating from the bladder mucosa and may develop in association with a variety of infectious and noninfectious conditions [1]. Although hematuria is not uncommon in childhood, gross hematuria occurs far less frequently than microscopic hematuria and generally requires a more extensive diagnostic evaluation [2]. A fundamental step in the assessment is distinguishing glomerular from non-glomerular hematuria, as this determines subsequent diagnostic pathways. In practice, this often requires a combination of urine microscopy and a comprehensive approach, helping narrow the differential diagnosis [3]. In pediatric patients, HC is most often encountered following chemotherapy, radiation therapy, or viral infections, where urothelial injury and microvascular damage are well-established pathogenic mechanisms [1, 4]. By contrast, cases occurring in otherwise healthy children without an identifiable precipitating factor are uncommon and remain insufficiently characterized in the literature.

Recent advances in understanding bladder inflammatory disorders have highlighted the protective role of the glycosaminoglycan (GAG) layer covering the urothelial surface. Disruption of this barrier has been associated with increased urothelial permeability, facilitating the penetration of urinary solutes into the submucosa and triggering local inflammation and tissue injury [5, 6]. Based on this concept, intravesical administration of GAG-replenishing agents, particularly hyaluronic acid (HA), has emerged as a therapeutic option aimed at restoring urothelial integrity and promoting mucosal healing [6–8]. While clinical experience in children remains limited, available reports suggest a favorable safety profile and encouraging outcomes in selected bladder disorders [9].

We report a case of noninfectious HC in a healthy 5-year-old boy successfully treated with intravesical HA. The case illustrates the challenges associated with the evaluation of unexplained gross hematuria in children. It supports the rationale for urothelial barrier restoration as a potential therapeutic strategy in carefully selected patients.

## Case report

**Figure 1:**
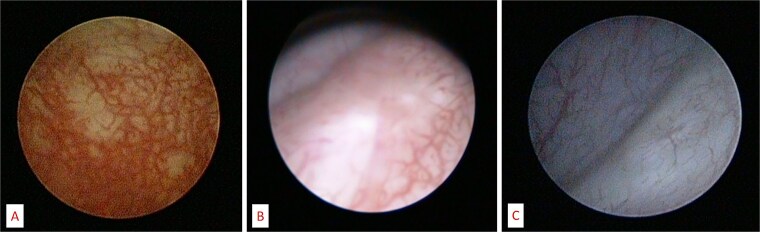
A–C. (A) Initial cystoscopy showed a diffusely inflamed bladder mucosa with marked erythema and patchy hemorrhagic areas. The urothelium appeared fragile, with increased vascular visibility; however, no focal lesions or structural abnormalities were observed. (B) At three months, repeat cystoscopy demonstrated clear improvement. Most of the inflammatory and hemorrhagic changes were resolved, with only mild residual erythema in the trigonal region. The left ureteral orifice was well visualized, and no active bleeding was present. (C) At seven months, cystoscopic findings were near normal. The bladder mucosa appeared smooth and intact, with complete resolution of previously observed changes and no signs of residual inflammation or bleeding.

A previously healthy 5-year-old boy was referred for evaluation of painless gross hematuria. There was no history of recent infection, trauma, medication use, chemotherapy, or radiation exposure. There was no history of urinary urgency, frequency, daytime incontinence, nocturnal enuresis, constipation, or other symptoms suggestive of bowel or bladder dysfunction. He appeared well on examination, with normal vital signs and no abnormal findings on physical assessment. Laboratory workup, including a complete blood count, inflammatory markers, renal function tests, and serum electrolytes, was within normal limits. Urinalysis confirmed significant hematuria in the absence of proteinuria, casts, or leukocyturia. Over the following two months, the episodes recurred intermittently, prompting referral to a tertiary center for further evaluation. The initial diagnostic approach was directed toward identifying the source of bleeding and excluding common causes. The absence of proteinuria, urinary casts, and leukocyturia made a glomerular or infectious origin unlikely. Repeated urine cultures remained sterile, and targeted viral testing, including cytomegalovirus and adenovirus, was negative. Initial renal and bladder ultrasonography demonstrated normal renal morphology and bladder appearance, without evidence of stones, masses, hydronephrosis, or bladder wall abnormalities. Further imaging studies were undertaken to exclude structural abnormalities or obstruction. Voiding cystourethrography showed no evidence of vesicoureteral reflux, while magnetic resonance urography did not reveal any structural pathology. In addition, static renal scintigraphy demonstrated preserved bilateral renal function without signs of renal scarring. Given the persistence of hematuria despite an otherwise unrevealing evaluation, cystoscopy was performed. This demonstrated diffuse mucosal hyperemia with focal hemorrhagic changes involving the proximal urethra, bladder neck, and posterior bladder wall (Fig. 1A), consistent with noninfectious HC, likely idiopathic in origin (Droller grade 2+) [7]. Bladder biopsy was not performed because cystoscopy revealed diffuse inflammatory changes without focal lesions or suspicious findings requiring histopathological evaluation.

In the absence of an identifiable cause and with ongoing symptoms, treatment was directed toward restoring the integrity of the urothelial barrier. HA (80 mg/50 mL) was administered through a sterile transurethral catheter in an outpatient setting once weekly for four consecutive weeks. The instilled solution was retained in the bladder for approximately 60 minutes whenever possible before spontaneous voiding. The treatment schedule was adapted from previously published intravesical HA protocols [8]. The response was prompt: gross hematuria resolved after the first instillation, with only transient microscopic hematuria thereafter. The treatment was well tolerated, and no adverse events were observed. Follow-up cystoscopy at three months demonstrated clear improvement, with marked regression of mucosal changes. By seven months, the bladder mucosa appeared nearly normal (Figs. 1B and C). At 12 months, the patient remained asymptomatic, with no recurrence of hematuria.

## Discussion

Persistent hematuria in children requires a structured yet flexible diagnostic approach, with early differentiation between glomerular and non-glomerular sources serving as a key first step. Although microscopic hematuria may be detected in up to 4% of school-aged children, persistence on repeated testing is considerably less common, occurring in approximately 0.25% of cases [2]. In contrast, gross hematuria is relatively uncommon and typically prompts more extensive evaluation. In the present case, the absence of proteinuria, dysmorphic erythrocytes, renal dysfunction, and inflammatory markers made a glomerular origin unlikely, while repeatedly sterile urine cultures and unremarkable imaging findings effectively excluded infectious and structural causes. In such situations, the diagnosis is often reached by exclusion rather than direct confirmation, supporting the interpretation of noninfectious HC, most likely idiopathic in origin [1, 3]. Within this diagnostic context, the decision to initiate intravesical HA was guided by the absence of an identifiable cause and the persistence of symptoms despite conservative measures. Taken together, these factors, in our view, justified a more targeted, mechanism-based therapeutic approach.

HC in children is most encountered in well-defined settings, such as chemotherapy, radiation therapy, or viral infections, where urothelial injury and microvascular damage are well recognized [1, 4, 9]. Outside these contexts, however, cases occurring in otherwise healthy children without identifiable triggers remain exceptionally rare. This not only underscores the unusual nature of the present case but also highlights the broader challenge of recognizing idiopathic forms of HC in everyday clinical practice. In our patient, the sustained remission observed over 12 months further supports the clinical relevance of this presentation. Cystoscopy, in this setting, proved especially informative, demonstrating diffuse mucosal inflammation and hemorrhagic changes while excluding focal pathology such as neoplasms or vascular malformations, thereby reinforcing the working diagnosis.

At a mechanistic level, increasing attention has been directed toward the role of urothelial barrier dysfunction in bladder inflammation. The GAG layer forms a protective interface over the bladder epithelium, and its disruption has been associated with increased permeability, allowing urinary solutes to penetrate the submucosa and trigger inflammation, vascular fragility, and bleeding [5, 6, 10, 11]. While this mechanism is well established in treatment-related cystitis, similar pathways have been proposed in idiopathic and inflammatory bladder conditions, offering a plausible explanation for cases such as ours.

From a therapeutic standpoint, management of HC is largely supportive in milder cases; however, persistent or recurrent symptoms often necessitate a more targeted approach [1]. In this context, intravesical therapies can be broadly divided into those aimed at achieving hemostasis and those intended to restore urothelial integrity. Among the latter, GAG-replenishing agents such as HA and chondroitin sulfate are particularly appealing, as they address the underlying barrier defect rather than simply mitigating the downstream consequences of mucosal injury [5, 10, 11].

Evidence supporting the use of intravesical HA is more robust in adult populations, particularly in interstitial cystitis/bladder pain syndrome and treatment-related HC, where it has been associated with both symptomatic improvement and restoration of urothelial barrier function [5, 10, 11]. Although study designs and treatment regimens vary, the overall body of evidence points toward a favorable safety profile and sustained clinical benefit in selected patients. Intravesical HA is generally considered safe, with reported adverse events being uncommon and usually mild, including transient dysuria, urinary urgency, bladder discomfort, or urinary tract infection associated with catheterization [12]. Serious treatment-related complications have rarely been reported [12]. By comparison, pediatric experience remains limited, with most available data derived from post-transplant settings, where intravesical therapies have shown encouraging results and acceptable tolerability [4, 9]. Outside these contexts, evidence is sparse, and reports involving otherwise healthy children are exceedingly rare, making it difficult to draw firm conclusions or establish standardized treatment protocols.

Against this background, the clinical course observed in our patient is particularly noteworthy. The rapid resolution of gross hematuria following the first instillation, together with sustained remission and progressive endoscopic normalization, suggests not only symptomatic improvement but also meaningful mucosal recovery. While a spontaneous course cannot be entirely excluded, the close temporal relationship between treatment initiation and clinical response supports a likely therapeutic effect. Taken together, this case highlights the potential role of urothelial barrier restoration as a rational, mechanism-based strategy in children with noninfectious HC, particularly when conventional causes have been carefully excluded. However, the limitations inherent to a single case report should be acknowledged. Although the temporal association between treatment initiation and clinical improvement suggests a therapeutic effect, spontaneous remission cannot be ruled out definitively. Larger multicenter and prospective studies are needed to evaluate efficacy better, identify patients most likely to benefit from this approach, and establish standardized treatment protocols in the pediatric population.
